# Ellagic Acid, the Active Compound of *Phyllanthus urinaria*, Exerts *In Vivo* Anti-Angiogenic Effect and Inhibits MMP-2 Activity

**DOI:** 10.1093/ecam/nep207

**Published:** 2011-01-11

**Authors:** Sheng-Teng Huang, Chen-Yu Wang, Rong-Chi Yang, Hsiao-Ting Wu, Su-Hui Yang, Yung-Chi Cheng, Jong-Hwei S. Pang

**Affiliations:** ^1^Department of Chinese Medicine, Chang Gung Memorial Hospital-Kaohsiung Medical Center, Chang Gung University College of Medicine, Taiwan; ^2^Department of Pharmacology, Yale University School of Medicine, New Haven, CT 06510, USA; ^3^School of Pharmacy, College of Medicine, National Taiwan University, Taipei 106, Taiwan; ^4^Chinese Herbal Pharmacy, Chang Gung Memorial Hospital, Tao-Yuan 333, Taiwan; ^5^Graduate Institute of Clinical Medical Sciences, Chang Gung University, Tao-Yuan 333, Taiwan

## Abstract

This study aimed to assess the potential anti-angiogenic mechanism of *Phyllanthus urinaria * (*P. urinaria*) and characterize the major compound in *P. urinaria* that exerts anti-angiogenic effect. The water extract of *P. urinaria* and Ellagic Acid were used to evaluate the anti-angiogenic effect in chorioallantoic membrane (CAM) in chicken embryo and human vascular endothelial cells (HUVECs). The matrix metalloproteinase-2 (MMP-2) activity was determined by gelatin zymography. The mRNA expressions of MMP-2, MMP-14 and tissue inhibitor of metalloproteinase-2 (TIMP-2) were analyzed by reverse transcription polymerase chain reaction (RT-PCR). Level of MMP-2 proteins in conditioned medium or cytosol was determined by western blot analysis. We confirmed that *P. urinaria's in vivo* anti-angiogenic effect was associated with a reduction in MMP-2 activity. Ellagic acid, one of the major polyphenolic components as identified in *P. urinaria* by high performance liquid chromatography mass spectrometry (HPLC/MS), exhibited the same anti-angiogenic effect *in vivo*. Both *P. urinaria* and Ellagic Acid inhibited MMP-2 activity in HUVECs with unchanged mRNA level. The mRNA expression levels of MMP-14 and TIMP-2 were not altered either. Results from comparing the change of MMP-2 protein levels in conditioned medium and cytosol of HUVECs after the *P. urinaria* or Ellagic Acid treatment revealed an inhibitory effect on the secretion of MMP-2 protein. This study concluded that Ellagic Acid is the active compound in *P. urinaria* to exhibit anti-angiogenic activity and to inhibit the secretion of MMP-2 protein from HUVECs.

## 1. Introduction

*Phyllanthus urinaria*, one of the herbal plants belonging to the genus *Phyllanthus* (Euphorbiaceae), is widely distributed in China, Southern India and South America. It has been used clinically in Asia to treat diarrhea, dysentery, hepatitis, edema, infantile malnutrition, acute conjunctivitis, aphthae and antibiotic resistant pyogenic infections [1, 2]. In our previous studies, water extracts prepared from *P. urinaria* were found to be cytotoxic to several tumor cell lines and the effects were mediated through the induction of apoptosis as the result of the up-regulation of Fas, FasL and Bax gene expression and the downregulation of Bcl-2 gene expression [3–5]. Cell culture studies also demonstrated that endothelial cell migration and matrix-induced tube formation in human vascular endothelial cell (HUVEC) system were inhibited [6]. We also reported the anti-angiogenic effect of *P. urinaria* in mice bearing Lewis lung carcinoma [6]. Various polyphenolic compounds with antioxidant effect have been identified in *P. urinaria* [7]; however, which polyphenolic compound may potentially contribute to the anti-angiogenic effect of *P. urinaria* is not clear.

Angiogenesis, an important aspect necessary for tumor development, proceeds through a series of steps including the early degradation of extracellular matrix (ECM) predominantly by matrix metalloproteinases (MMPs), migration and proliferation of vascular endothelial cells, and maturation of the new blood vessel in response to local factors [8]. MMPs are a growing family of Zn-dependent matrix proteases, which are key mediators of ECM remodeling. MMPs not only are involved in the removal of structural proteins in the ECM, but also indirectly control multiple cellular functions including cell growth, apoptosis, angiogenesis, invasion and metastasis through its proteolytic action on growth factors, cell adhesion molecules and other bioactive proteins [9].

The present study demonstrated that *P. urinaria* could reduce MMP-2 activity in chick chorioallantoic membrane (CAM) assay and in HUVECs, which was one potential mechanism for its anti-angiogenic effect. The key active compound in the *P. urinaria* extract, which exerts its anti-angiogenic effect, was also identified.

## 2. Methods

### 2.1. Preparation of *P. urinaria* Extracts

*P. urinaria* used in this study was identified based on the definition described in Flora of Taiwan [10] and authenticated by Dr Rong-Chi Yang, the chief of the Chinese Herbal Pharmacy in Chang Gung Memorial Hospital. A sample of *P. urinaria* is deposited in set D, TN: No 173156 in the herbarium of National Taiwan University. The extract was prepared according to Taiwanese good manufacturing practice (GMP) methodologies and guidelines. Briefly, the whole plant was minced and extracted with boiling water 1 : 20 (w/v) for 4 h; then, the extract was removed and water, 1 : 20 (w/v), was again added and boiled for 4 h. The resulting crude extract was filtered and lyophilized down to a dry powder. The extract used in the experiments was prepared by dissolving the powder in sterile water at the desired concentration and storing at −20°C. The extraction rate was 26.4%, that is, 1 gm of lyophilized powder is equal to 3.79 gm original herb.

### 2.2. Mass Spectrometry

High performance liquid chromatography (HPLC) was performed on Shimadzu SIL-20A LITE (Shimadzu Corporation, Columbia, MD, USA). Shimadzu SPD-10AVP UV-Vis Detector was used at **λ** = 270 nm. The chromatographic separation was carried out on an Agilent Zorbax Eclipse XD8-C18 column (4.6 × 150 mm i.d.; 3.5 *μ*m particle size) eluted with the mixture of 0.01% trifluoroacetic acid_(aq)_ (A) and acetonitrile (B). The linear gradient program was set from 95 : 5 (A : B, v/v) to 80 : 20 in 22 min, then the concentration of acetonitrile was increased from 20 to 30% in the following 28 min. Flow rate was 0.6 mL min^−1^. Sample injecting volume was 30 *μ*l each. Mass spectra were acquired using a LTQ XL Mass Spectrometer (Thermo Electron Corp, San Jose, CA, USA) equipped with an electrospray ionization source operated in the negative mode using the following conditions: Spray voltage (4.5 kV); heated capillary temperature (245°C); capillary voltage 47 V; tube lens offset (5.25 V). Nitrogen was used as the sheath and auxiliary gas at 35 and 10 units, respectively. Mass spectrometry (MS^n^) experiments were carried out using Helium as collision gas. Data acquisition and analysis were accomplished with Xcalibur software version 2.0 (Thermo Electron Corp.). Gallic acid and Ellagic Acid (Sigma-Aldrich Corporation, WI, USA) were used as reference compounds and dissolved separately in methanol (MeOH).

### 2.3. Glucuronidation Metabolites of *P. urinaria* Extract

The glucuronidation metabolites of water extract of *P. urinaria* were measured by treating with *β*-glucuronidase for glucuronide hydrolysis. An aliquot of 100 *μ*L *P. urinaria* extract (100 mg mL^−1^) was titrated with HCl (10 N) to pH 1-2 and incubated for 1 h at 37°C and again titrated with NaOH (10 N) back to pH 6.8. An aliquot of 1 *μ*L of *β*-glucuronidase (134.60 units mL^−1^) was added into a portion of 20 *μ*l *P. urinaria* extract and 80 *μ*L Tris (100 mM, pH 6.8) and then incubated at 37°C for 2 h. Control incubations in the presence and absence of HCl without *β*-glucuronidase were performed simultaneously. After incubation, a 3-fold volume of acetonitrile/methanol (2 : 1) was added to the samples followed by vortex mixing, centrifugation and filtration. The filtrate (20 *μ*L) was injected onto the HPLC system.

### 2.4. Cell Culture

HUVECs were isolated from the vein of human umbilical cords and grown in EGM provided by Clonetics (MD, USA). Cells were maintained in a humidified atmosphere with 5% CO_2_/95% air at 37°C and passaged 3–5 times prior to use in experiments. To examine the effect of *P. urinaria* extract on cell function, cells at 80–90% confluency were treated with 0–2 mg mL^−1^ of *P. urinaria* extract or 0–10 *μ*M Ellagic Acid for 24 h.

### 2.5. CAM Angiogenesis Aassay

Angiogenesis assays were performed on the CAMs of 10-day-old chick embryos with minor modifications [11]. First, a small hole was made through the shell at the air sac end of the chick egg using a small craft drill. A second hole was drilled on the broad side of the egg directly over embryonic blood vessels. Negative pressure was applied to the original hole, resulting in the CAM pulling away from the shell membrane, creating a false air sac. A window was cut through the shell over the dropped CAM and saturated with 100 *μ*L of sterile 1 × PBS (phosphate buffer solution) containing 120 ng phorbol-12-myristate-13-acetate (PMA) in the presence or absence of *P. urinaria* or Ellagic Acid. Sterile 1 × PBS was used for the control egg. The shells were covered with adhesive tape and incubated in a 37°C incubator. After 48 h, the CAM tissues were harvested and examined for angiogenesis under a stereomicroscope (Zeiss, Germany). The angiogenic index was defined as the mean number of visible blood vessel branch points within the defined area of the membrane. Photographs were taken at 16x magnification.

### 2.6. Zymography Analysis

CAM harvested from the egg was homogenized in 1 × PBS. The supernatant was collected after brief centrifugation and used for the analysis of MMP activity. Conditioned media collected from cultured human endothelial cells with 24-h treatment with *P. urinaria* or Ellagic Acid were also processed for analysis of their influence on MMP activity. Protein samples were loaded onto 10% sodium dodecyl sulfate polyacrylamide gel electrophoresis (SDS-PAGE) gels in which 1% gelatin (Amersham Life Science, Cleveland, OH, USA) was incorporated [12]. After migration, gels were incubated with 2.5% Triton-X 100 twice for 30 min at room temperature, washed for 5 min in TNCA (50 mM Tris pH 7.5, 200 mM NaCl, 5 mM CaCl_2_) and further incubated for 16 h in TNCA in a shaking bath at 37°C. Gels were stained for 1 h in coomassie blue (0.1% coomassie brillant blue R-250, 50% methanol, 10% acetic acid) and destained in 5% methanol/9% acetic acid until proper contrast was achieved. White bands on blue background indicated zones of digestion corresponding to the presence of different MMPs.

### 2.7. RNA Isolation and Reverse Transcription Polymerase Chain Reaction

Total cellular RNA was isolated by lysis of cells in a guanidinium isothiocyanate buffer, followed by single step phenol-chloroform-isoamyl alcohol extraction procedure modified from that previously described [13]. Briefly, *P. urinaria* or Ellagic Acid of untreated or treated cells were harvested and lysed in 4 M guanidinium isothiocyanate, 25 mM sodium citrate (pH 7.0), 0.5% sodium sarkosine and 0.1 M *β*-mercaptoethanol. Sequentially, 1/10 volume of 2 M sodium acetate (pH 4.0), one volume of phenol and 1/5 volume of chloroform-isoamyl alcohol (49 : 1, v : v) were added to the homogenate. After vigorous vortexing for 30 s, the solution was centrifuged at 10 000 g for 15 min at 4°C. After removal of the aqueous phase, RNA was precipitated by the addition of 0.5 mL isopropanol. One *μ*g of total RNA was reverse-transcribed into cDNA by incubating with 200 units of reverse transcriptase in 20 *μ*L of reaction buffer containing 0.25 *μ*g of random primers and 0.8 mM dNTPs at 42°C for 60 min. Two microliters of cDNA was used as template for the PCR reaction. PCR was performed in buffer containing 10 mM Tris, pH 8.3, 50 mM KCl, 1.5 mM MgCl_2_, 0.2 mM dNTPs, 1 *μ*M of each primer and 5 units Taq DNA polymerase for 30 cycles of denaturation at 94°C for 1 min, annealing at 55°C for 1 min and extension at 72°C for 2 min. The resulting PCR product was analyzed by ethidium bromide (EtBr) stained 1.5% agarose gel electrophoresis. Sequences for the specific primers used in the PCR are MMP-2 forward primer (5′-GTTTCCATTCCGCTTCCAGG-3′) and reverse primer (5′-TGCCCTTGATGTCATCCTGG-3′); MMP-14 forward primer (5′-ACATACGAGGCCATTCGCAA-3′) and reverse primer (5′-TCCTTGAAGACAAACATCTCCCC-3′); TIMP-2 forward primer (5′-ACAGGCGTTTTGCAATGCAG-3′) and reverse primer (5′-CGCGCAAGAACCATCACTTC-3′); GAPDH forward primer (5′-TTCATTGACCTCAACTACAT-3′) and reverse primer (5′-GAGGGGCCATCCACAGTCTT-3′).

### 2.8. Western Blotting

Protein concentrations were determined by the Bradford method (Bio-Rad, CA, USA). Samples with equal amount of proteins were subjected to 10% SDS-PAGE and transferred onto a polyvinylidene difluoride (PVDF) (Millipore, Bedford, MA, USA) membrane. The membrane was incubated at room temperature in blocking solution (1% bovine serum albumin (BSA), 1% goat serum in PBS) for 1 h, followed by 2 h incubation in blocking solution containing an appropriate dilution (1 : 1000) of primary antibody, for example, anti-MMP-2 antibody (NeoMarkers, Fremonk, CA, USA). After washing, the membrane was incubated in PBS containing goat anti-mouse IgG conjugated with horseradish peroxidase (Sigma, St Louis, MO, USA) for 1 hour. The membrane was washed and the positive signals were developed with chemiluminescence reagent (Amersham Pharmacia Biotech, Little Chalfont Buckinghamshire, England). Membrane was exposed to Fuji medical X-ray film (Fuji Ltd, Tokyo, Japan) for 30 min.

### 2.9. Statistical Aanalysis

All statistical analyses were performed using SigmaStat statistical software (version 2.0, Jandel Scientific, CA, USA). Results were represented as means ± standard deviation (SD). ANOVA was carried out when multiple comparisons were evaluated. Values were considered to be significant at *P* < .05. All experiments were repeated at least three times independently.

## 4. Results

### 4.1. *P. urinaria* Extract Inhibited In Vivo Angiogenesis and MMP-2 Activity on CAMs of Chicken Embryos

**Figure 1:**
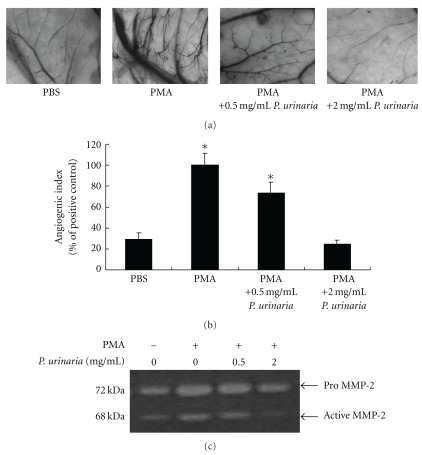
*Phyllanthus urinaria* extract inhibited the *in vivo* angiogenesis and MMP-2 activity induced on the CAMs of chicken embryos. (a) Representative photos of CAMs at the end of experiment. (b) Angiogenic index was quantified by counting the number of blood vessel branch points on each CAM. (c) Decreased MMP-2 activity in *P. urinaria* treated chorioallantoic memebranes. The protein extract was prepared by homogenizing the CAMs, centrifuging and using the resulting supernatant for gelatin zymography analysis. Bar value is the mean ± SD of three independent experiments. Asterisk means statistic significance in comparison with the vehicle control.

We first used the CAM assay to study the *in vivo* effect of *P. urinaria* extract on PMA-induced angiogenesis. As shown in Figure 1(a), the vessels in the control CAM treated with 1 × PBS only were small and less branched than the newly spouting vessels induced by PMA under stereomicroscopic observation. The angiogenic response was effectively inhibited by the addition of *P. urinaria* extract. Addition of 0.5 and 2 mg mL^−1^ of *P. urinaria* extracts lead to a decrease of 24 and 74% in the angiogenic index as determined by counting the number of blood vessel branch points (Figure 1(b)). These results confirm the inhibitory effect of *P. urinaria* extract on *in vivo* angiogenesis. To further investigate the underlying molecular mechanism, we performed gelatin zymography to analyze the MMP activity in protein samples prepared from CAMs after *P. urinaria* treatment. The dominant gelatin-type of MMP from CAMs was MMP-2, whereas MMP-9 was minimally present. PMA could induce MMP-2 activity and correlated well with the induction of angiogenesis. In the presence of aqueous extract of *P. urinaria,* PMA-induced MMP-2 activity was suppressed in a dose-dependent manner (Figure 1(c)).

### 4.2. Chemical Chromatography of the Aqueous Extract of *P. urinaria*

**Figure 2:**
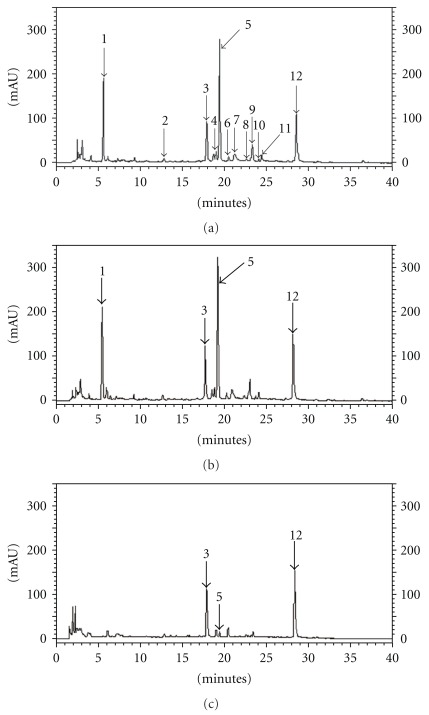
HPLC chromatograms of (a) *P. urinaria* water extract and (b) *P. urinaria* water extract treated with HCL alone or (c) with HCL following *β*-glucuronidase treatment monitored at 270 nm.

**Figure 3:**
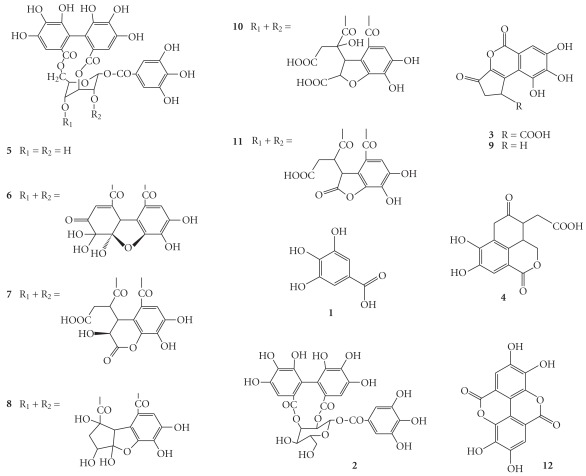
Structures of the identified compounds in *P. urinaria* extract.

Analysis of the water extracts of *P. urinaria* by HPLC led to the identification of twelve compounds as shown in Figure 2(a). The mass structures of these compounds were tentatively assigned based on their mass data mining from existing literature. The mass of peak 1 (*R_t_* at 5.8 min) was determined to be 170 by liquid chromatography electrospray ionisation tandem mass spectrometry (LC/(−)ESI-MS), yielding [M–H]^−^ at *m/z* 169. The major fragment was shown at *m/z* 151 (M–H–H_2_O) and 125 (M–COOH), consistent with the structure of gallic acid (Figure 3) [14]. The mass of peak 3 (*R*_*t*_ at 18.0 min) and peak 9 (*R*_*t*_ at 23.3 min) were determined to be 292 and 248, yielding [M–H]^−^ at *m/z* 291 and 247, respectively. The major fragment of peak 3 was shown at *m/z* 247 (M–COOH), and the minor fragment ions were the same as peak 9. Thus, the determined mass of peak 3 is 44 higher than peak 9, suggesting a carboxylic acid derivative of peak 9. By searching the bioactive components previously found in *Phyllanthus* species [14, 15], peak 3 and peak 9 were assigned to be brevifolin carboxylic acid and brevifolin, respectively (Figure 3). LC/(−)ESI-MS analysis of peak 4 (*R*_*t*_ at 19.0 min) produced the same [M–H]^−^ at *m*/*z* 291 with peak 3. Collision activated decomposition mass spectrum [(−)CAD-MS/MS] analysis of peak 4 yielded intense product ions at *m*/*z* 247 (M–COOH), 203 (M–2COOH). Compared with the mass and mass fragments of compounds found in other *Phyllanthus* species, the structure of peak 4 was assigned to be phyllanthusiin E (Figure 3) [16]. LC/(−)ESI-MS analysis of peak 5 (*R*_*t*_ at 19.5 min), peak 6 (*R*_*t*_ at 20.5 min), peak 7 (*R*_*t*_ at 21.2 min), peak 8 (*R*_*t*_ at 22.8 min), peak 10 (*R*_*t*_ at 24.2 min) and peak 11 (*R*_*t*_ at 24.6 min) yielded [M–H]^−^ at *m/z* 633, 951, 953, 925, 969 and 924, respectively. (−)CAD-MS/MS analysis of all these components yielded intense product ions at *m/z* 633 and 301, resulting from the loss of the hexahydroxydiphenoyl and/or galloyl moiety fragments [17]. These ions are significant fragments of ellagitannins. After comparing the mass and other minor fragment ions with tannins found in other *Phyllanthus* species, the structures of these peaks were assigned to be corilagin [14, 17], geraniin [18], chebulagic acid [19], phyllanthusiin C [20], phyllanthusiin B [20] and phyllanthusiin U [14], respectively (Figure 3). Peak 2 (*R*_*t*_ at ∼14.0 min) produced a same [M–H]^−^ at *m/z* 633 with peak 5. The fragment ions at *m/z* 453 (M-H-Gal) and 301 (M-H-Gal-glc), corresponding to peak 5, are consistent with the structure of isostrictiniin [21]. Peak 12 (*R*_*t*_ at 28.7 min) yielded a [M–H]^−^ at *m/z* 301, consistent with a mass of 302. The same fragment pattern was shown by comparing (^−^)CAD-MS/MS analysis of peak 12 with the product ion spectral analysis of the fragment ion at *m/z* 301 (MS^3^) in peak 5 (corilagin). The information suggested an Ellagic Acid moiety and compared with the fragments with literature [14], peak 12 was assigned to be Ellagic Acid (Figure 3). The identities of peaks 1 and 12 were further confirmed by chemical markers (Mass and its HPLC retention times) to be gallic and Ellagic Acid, respectively. Based on standard curve of mass unit versus the amount of standards, the amounts of gallic and Ellagic Acid found in *P. urinaria* in the present study were 3.09 ± 0.17 and 2.67 ± 0.16 mg g^−1^ DW, respectively.

After pH 1-2 treatment of *P. urinaria*, to mimic gastric pH, there were no specific changes (Figure 2(b)) in the chemical profile compared to untreated *P. urinara* (Figure 2(a)). Since intestine and colon have anaerobic bacteria, which have *β*-glucuronidase activity capable of digesting glucuronides found in herbal extract, *P. urinaria* aqueous extract was further treated with *E. coli* *β*-glucuronidase. There was substantial change of chemical fingerprint of *P. urinaria*. The amounts of compound 3 and 12 (Ellagic Acid) were not altered much (Figure 2(c)). Meanwhile, gallic acid was hydrolyzed completely after neutralizing to pH 6.8 using NaOH from pH1-2 treatment and incubated at a temperature of 37°C (data not shown).

### 4.3. Ellagic Acid In Vivo Inhibited Angiogenesis and MMP-2 Activity on CAMs of Chicken Embryos

**Figure 4:**
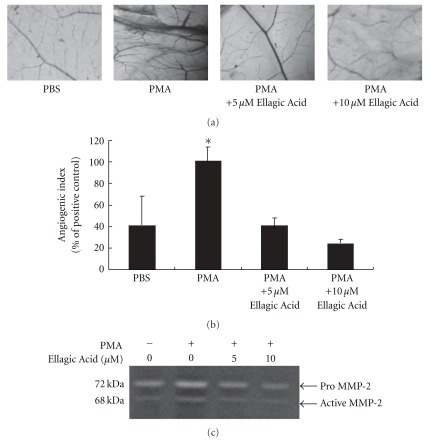
Ellagic Acid inhibited the *in vivo* angiogenesis and MMP-2 activity induced on the CAMs of chicken embryos. (a) Representative photos of CAMs at the end of experiment. (b) Angiogenic index was quantified by counting the number of blood vessel branch points on each CAM. (c) Decreased MMP-2 activity in Ellagic Acid treated chorioallantoic memebranes. The protein extract was prepared by homogenizing the CAMs, centrifuging and using the resulting supernatant for gelatin zymography analysis. Bar value is the mean ± SD of three independent experiments. Asterisk means statistic significance in comparison with the vehicle control.

Ellagic acid, being resistant to pH 1-2 and *E. coliβ*-glucuronidase treatment, was shown by others to inhibit angiogenesis *in vitro* [22]. We therefore examined the effect of Ellagic Acid in comparison with aqueous extract of *P. urinaria* by CAM assay. As shown in Figure 4(a), PMA-induced angiogenesis was effectively inhibited by the addition of Ellagic Acid. Addition of 5 and 10 *μ*M Ellagic Acid resulted in a decrease of the angiogenic index by 62 and 83%, respectively (Figure 4(b)). Additionally, gelatin zymography demonstrated that MMP-2 activity induced by PMA from CAM was also inhibited (Figure 4(c)) by Ellagic Acid in a dose-dependent manner. The potency of Ellagic Acid is equivalent to its amount in *P. urinaria* aqueous extract. 

### 4.4. Inhibition of MMP-2 Activity, but Not MMP-2, 14 and Tissue Inhibitor of Metalloproteinase-2 Gene Expression by *P. urinaria* and Ellagic Acid *In Vitro*

**Figure 5:**
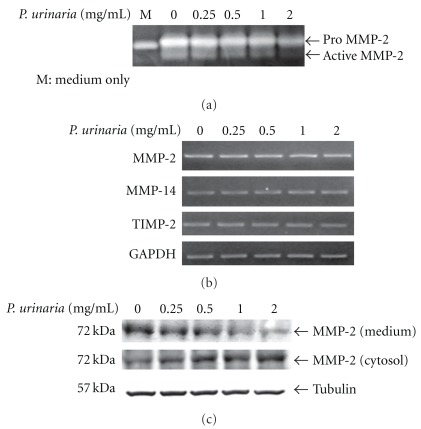
(a) *Phyllanthus urinaria* extract dose-dependently inhibited the MMP-2 activity in HUVECs. Conditioned media taken from cultures of HUVECs, with or without the treatment of *P. urinaria* extract for 24 h, were used for the gelatin zymography analysis. To study the effects of *P. urinaria* extract on the mRNA expression of MMP in HUVECs, cells were treated with (b) *P. urinaria* for 24 h and the mRNA expressions of MMP-2, MMP-14, TIMP-2 and GAPDH were determined by RT-PCR analysis. (c) Cells treated with *P. urinaria* for 24 h were processed for western blotting to analyze the protein levels of MMP-2 in the conditioned media and cytosol.

**Figure 6:**
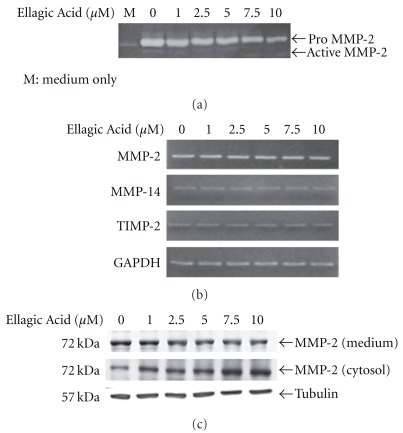
(a) Ellagic Acid dose-dependently inhibited the MMP-2 activity in HUVECs. Conditioned media taken from cultures of HUVECs, with or without the treatment of Ellagic Acid for 24 h, were used for the gelatin zymography analysis. To study the effects of Ellagic Acid on the mRNA expression of MMP in HUVECs, cells were treated with (b) Ellagic Acid for 24 h and the mRNA expressions of MMP-2, MMP-14, TIMP-2 and GAPDH were determined by RT-PCR analysis. (c) Cells treated with Ellagic Acid for 24 h were processed for western blotting to analyze the protein levels of MMP-2 in the conditioned media and cytosol.

To further examine the potential mechanism underlying the anti-angiogenic effect of *P. urinaria* in an *in vitro* cell model, we tested the effect of *P. urinaria* on MMP-2 activity in HUVECs. Results from gelatin zymography revealed that the inhibitory effect of *P. urinaria* or Ellagic Acid on MMP-2 activity also occurred in cultures of HUVECs (Figures 5(a) and 6(a)) dose-dependently with the potency of Ellagic Acid about the same as that in aqueous extract of *P. urinaria*.

It is known that multiple regulatory mechanisms are involved in the modulation of MMP-2 expression and activity. We examined whether *P. urinaria* or Ellagic Acid could affect the MMP-2 gene expression at the transcriptional level. As shown in Figures 5(b) and 6(b), the expression of MMP-2 mRNA remained unchanged after the *P. urinaria* or Ellagic Acid treatment. The other two mRNAs, including MMP-14 and TIMP-2, known to play important roles in regulating the MMP-2 activity, were also not altered by *P. urinaria* or Ellagic Acid treatment either.

### 4.5. Inhibition of MMP-2 Secretion in HUVECs by *P. urinaria* and Ellagic Acid

The process of MMP-2 secretion to the extracellular environment where it actually functions could be another critical mechanism for the regulation of angiogenesis. As shown in Figures 5(c) and 6(c), the protein levels of MMP-2 in conditioned medium after *P. urinaria* or Ellagic Acid treatment were dose-dependently decreased and correlated well with the decrease of MMP-2 activity as analyzed by gelatin zymography. Interestingly, the protein levels of MMP-2 in the cytosol were conversely increased after *P. urinaria* or Ellagic Acid treatment in a dose-dependent manner (Figures 5(c) and 6(c)). The accumulation of MMP-2 protein in the cytosol and the decrease of MMP-2 protein in conditioned medium indicated that a target was involved in the inhibition of secretory pathway of MMP-2 by *P. urinaria* or Ellagic Acid.

## 5. Discussion

Angiogenesis, the formation of new blood vessels to supply enough nutrition and oxygen to a local environment [23], is known to play an important role in several physiological and pathological processes [24, 25]. The primary step in the angiogenic process relies on the degradation of subendothelial basement membrane and surrounding ECM proteins [26]. MMPs, which degrade ECM proteins, are involved in angiogenesis both *in vitro* [27] and *in vivo* [28]. Therefore, inhibition on the early degradation of ECM proteins predominantly by MMPs is considered an important strategy to inhibit angiogenesis. In this study, we attempted to further clarify potential mechanism underlying the anti-angiogenic effect of *P. urinaria,* which was discovered previously in our laboratory [6]. Results from the *in vivo* CAM assay showed that *P. urinaria* efficiently inhibited embryonic angiogenesis and the MMP-2 activity in the protein extract prepared from CAM. This suggested that the inhibition of angiogenesis in chicken embryo by *P. urinaria* could be a result, in part, from the failure of vascular endothelial cells to degrade the surrounding ECM and therefore impede the following migration and proliferation. Among the various MMPs (MMP-1, -2, -9, -13 and -14) produced by vascular endothelial cells including, MMP-2 and MMP-14 (MT1-MMP) have been linked to cell invasion and network formation [29, 30]. The inhibition of MMP-2 activity was shown to be capable of disrupting the tube formation of endothelial cells [31], which may also explain our previous finding that *P. urinaria* could inhibit the matrix-induced tube formation of vascular endothelial cells.

In recent years, extract from natural products such as propolis and bilberry [32, 33] exhibited anti-angiogenic effect through suppression of ERK1/2 phosphorylation. However, the exact compound from propolis and bilberry responsible for anti-angiogenesis is not clear. In order to further identify which compounds in *P. urinaria* extract exert their anti-cancer effect through angiogenic pathways, we characterized 12 polyphenolic compounds from *P. urinaria* that were recognized by HPLC and LC/MS. The major compound in *P. urinaria* was corilagin followed by gallic acid and Ellagic Acid. Herein, the fingerprint profile of *P. urinaria* can be used for plant identification and batch-to-batch quality control. In addition, we found that 8.33 ± 0.49 *μ*M of Ellagic Acid was equal to 1 mg mL^−1^ of *P. urinaria* in water extract as demonstrated by *in vivo* and *in vitro* anti-angiogenesis assays. Ellagic Acid (4,4′,5,5′6,6′-hexahydroxydiphenic acid 2,6,2′,6′-dilactone) is a natural phenolic constituent present in woody plants, berries, grapes and nuts [34]. Ellagic Acid has been reported to show many biological properties including antioxidant, antiproliferative and apoptosis-inducing activities [35]. Recently, the anticancer effect of Ellagic Acid on the angiogenic process was studied *in vitro* and found to inhibit migration and tube formation by downregulation of both PDGF and VEGF receptors [22]. In report by Losso et al. [36], they suggested that inhibition of cancer cell proliferation by Ellagic Acid could be mediated by regulation of MMPs, VEGF and induction of apoptosis in cancer cells, but not normal cells. According to the report of Talcott et al. [37], 5–10 *μ*mol L^−1^ of Ellagic Acid in cell culture could be a physiological concentration in the blood plasma after food consumption. In our study, we further confirm the observations of others. In chicken embryos treated with *P. urinaria* (1-2 mg mL^−1^) or Ellagic Acid (5–10 *μ*M), an anti-angiogensis effect was observed and could be due to the inhibition of MMP-2 activity. HUVECs exposed to similar *P. urinaria* or Ellagic Acid concentrations as the CAM demonstrated the same suppressive effect of MMP-2 activity in HUVECs. In a bioavailability study using wild-type mice [38], Ellagic Acid was detectable in the plasma level with oral feeding of ellagitannins to mice. In our study, digestive-mimicking treatments of HCl pH 1-2 followed by exposure to *β*-glucuronidase, effectively changed most compounds except for Ellagic Acid. This suggests Ellagic Acid will survive digestion and be available for absorption into circulation. From these studies, we conclude that the main active constituent of *P. urinaria* for its antiangiogenic activity *in vivo* is Ellagic Acid.

MMP-2 activity is known to be regulated at multiple levels [39]. The MMP-2 promoter is constitutively active in endothelial cells, which is seldom inducible by growth factors or cytokines, although some exceptions exist in other cell lines [40]. Our results showed that the inhibitory effect of *P. urinaria* or Ellagic Acid on MMP-2 activity was not at the transcriptional level. Another important regulatory mechanism for the activation of MMP-2, mediated by MMP-14, occurs mainly at the cell surface. Latent pro-MMP-2 is activated on the cell surface by MMP-14 in a process regulated by the tissue inhibitor of metalloproteinase (TIMP)-2 [41, 42]. Under physiological conditions, MMP-14, free of TIMP-2, can activate complex ternary pro-MMP-2. Since the ratio between the amounts of pro-MMP-2 and active MMP-2 as shown in zymography data did not change upon the treatment with either *P. urinaria* or Ellagic Acid. Both the amounts of pro-MMP-2 and active MMP-2 decreased in parallel and dose-dependently after drug treatment. It indicated that the activation of pro-MMP-2 toward active MMP-2 was not affected by *P. urinaria* and Ellagic Acid. There is, therefore, a close relationship between MMP-2 activation through the interactions of pro-MMP-2, MMP14 and TIMP-2. mRNA expression in vascular endothelial cells, however, was not altered after *P. urinaria* or Ellagic Acid treatment. Therefore, the anti-angiogenic effect of *P. urinaria* on vascular endothelial cells was most likely mediated by a mechanism other than MMP transcriptional regulation.

Because there exists a secretory function to allow rapid mobilization and utilization of these crucial MMP-2 regulatory enzymes in the early phase of angiogenesis [43], we focused on cellular secretions after *P. urinaria* or Ellagic Acid treatment. Results showed that MMP-2 protein level in the conditioned medium decreased dose-dependently in *P. urinaria* or Ellagic Acid treated HUVECs, while the level of MMP-2 proteins in the cells increased in a dose-dependent manner. This suggests that *P. urinaria* and Ellagic Acid can inhibit the secretion of MMP-2 in HUVECs. MMP-2 secretion itself is probably regulated by multiple pathways. The detail mechanism still needs further investigation.

**Figure 7:**
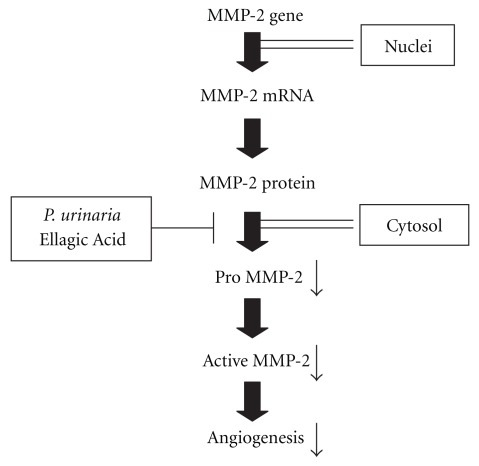
Schematic diagram illustrates the molecular mechanism that is responsible for the anti-angiogenic effect of *P. urinaria* and Ellagic Acid.

In conclusion as shown in Figure 7, a potent inhibitory effect of *P. urinaria* on MMP-2 activity, both *in vivo* and *in vitro*, which may be one of the mechanisms leading to the suppression of angiogenesis, was identified. The active compound of *P. urinaria* is Ellagic Acid. The mechanisms of extracellular suppression in MMP-2 activity go through the inhibition of MMP-2 secretion from cells. *P. urinaria* or Ellagic Acid could be considered for future clinical development as an antitumor agent. 

## Funding

Chang Gung Memorial Hospital (CMRPG33101).
